# The impact of the 21-gene assay on adjuvant treatment decisions in oestrogen receptor-positive early breast cancer: a prospective study

**DOI:** 10.1038/bjc.2016.48

**Published:** 2016-03-08

**Authors:** Anna Kuchel, Tim Robinson, Charles Comins, Mike Shere, Mohini Varughese, Geoff Sparrow, Ajay Sahu, Louise Saunders, Amit Bahl, Simon J Cawthorn, Jeremy P Braybrooke

**Affiliations:** 1 Bristol Cancer Institute, University Hospitals Bristol NHS Foundation Trust, Bristol BS2 8ED, UK; 2 Bristol Breast Care Centre, North Bristol NHS Trust, Bristol BS10 5NB, UK; 3 The Beacon Centre, Musgrove Park Hospital, Taunton and Somerset NHS Foundation Trust, Taunton TA1 5DA, UK; 4 Yeovil District Hospital NHS Foundation Trust, Yeovil BA21 4AT, UK

**Keywords:** Adjuvant chemotherapy, breast cancer, decisional conflict, Onco*type* DX, Recurrence Score

## Abstract

**Background:**

International guidelines, including NICE, recommend using the 21-gene Recurrence Score assay for guiding adjuvant treatment decisions in ER+, HER2-negative early breast cancer (BC). We investigated the impact of adding this assay to standard pathological tests on clinicians'/patients' treatment decisions and on patients' decisional conflict in the United Kingdom.

**Methods:**

In this prospective multicentre study, eligibility criteria included: ER+ HER2-negative BC (N0/Nmic for patients ⩽50 years; ⩽3 positive lymph nodes for patients >50 years) and being fit for chemotherapy. Physicians'/patients' treatment choices and patients' decisional conflict were recorded pre- and post testing.

**Results:**

The analysis included 137 patients. Overall, adjuvant treatment recommendations changed in 40.7% of patients, with the direction of the change consistent with the Recurrence Score results (net decrease in chemotherapy recommendation rate in low Recurrence Score patients and net increase in high Recurrence Score patients). Patients' choices were generally consistent with physicians' recommendations. Post-testing, patients' decisional conflict decreased significantly (*P*<0.0001). In the 67 patients meeting the NICE criteria for testing, the recommendation change rate was 49.3%.

**Conclusions:**

Recurrence Score testing significantly influenced treatment recommendations overall and in the subgroup of patients meeting the NICE criteria, suggesting that this test could substantially alter treatment patterns in the United Kingdom.

Adjuvant chemotherapy improves overall survival of patients with early-stage breast cancer (Early Breast Cancer Trialists' Collaborative Group, 2005). However, for individual patients with oestrogen receptor positive (ER+), human epidermal growth factor receptor 2 negative (HER2−) disease, the benefit of chemotherapy remains uncertain. The 21-gene Recurrence Score assay (Onco*type* DX, Genomic Health, Inc., Redwood City, CA, USA) is a validated real-time reverse transcription PCR (RT–PCR) assay that provides prognostic (10-year risk of distant recurrence) and predictive (the likelihood of benefit from chemotherapy) information for patients with ER+ HER2− early-stage invasive breast cancer treated with endocrine therapy (Paik *et al*, 2004; Habel *et al*, 2006; Paik *et al*, 2006; Goldstein *et al*, 2008; Albain *et al*, 2010; Dowsett *et al*, 2010; Toi *et al*, 2010; Mamounas *et al*, 2012; Sparano *et al*, 2015).

Since the 21-gene assay became available in 2004, more than 500 000 tests have been performed for patients in more than 70 countries (Genomic Health, data on file). The assay has been incorporated into major international guidelines such as the National Comprehensive Cancer Network, American Society of Clinical Oncology, St Gallen, and the European Society for Medical Oncology guidelines (Harris *et al*, 2007; Aebi *et al*, 2011; Goldhirsch *et al*, 2013; NCCN, 2015). In the United Kingdom, favourable National Institute for Health and Care Excellence (NICE) guidance (DG10) on using this test for patients with intermediate-risk early breast cancer has been available since September 2013. NICE defines intermediate risk as patients with lymph node-negative breast cancer with either a Nottingham Prognostic Index (NPI) >3.4 or intermediate risk defined by other decision making tools such as Adjuvant! Online or PREDICT (NICE, 2013). However, in many areas of the United Kingdom this guidance has not yet been implemented due to lack of reimbursement. Data on the impact of this test in the United Kingdom, overall, and in particular on the subpopulation of patients meeting the NICE criteria, are very limited.

Prospective decision impact studies conducted in European countries, the United States, Asian countries, and Australia have consistently demonstrated that the assay has a substantial impact on adjuvant treatment decisions leading to an overall reduction in chemotherapy use (Lo *et al*, 2010; Albanell *et al*, 2012; Davidson *et al*, 2012; de Boer *et al*, 2013; Eiermann *et al*, 2013; Holt *et al*, 2013; Yamauchi *et al*, 2014; Gligorov *et al*, 2015).

The current study was designed to evaluate whether, for patients with ER+ HER2− early breast cancer, adding the 21-gene assay to standard pathological tests would influence clinicians' and patients' treatment decisions as well as patients' decisional conflict in the United Kingdom.

## Materials and Methods

### Study design and patients

In this prospective multicentre study, patients were eligible if they had ER+ HER2− invasive breast cancer with negative axillary lymph nodes or micrometastases (for patients ⩽50 years) or with up to three positive axillary lymph nodes (for patients >50 years). In addition, patients' performance status (according to Eastern Cooperative Oncology Group (ECOG)) had to be 0 or 1, and patients had to be fit for chemotherapy. No exclusion criteria were applied with respect to tumour size or grade. All patients discussed at the post-surgical multidisciplinary team meeting at their respective institutions, who met the eligibility criteria and were suitable to receive chemotherapy as part of their adjuvant therapy were offered entry into the study. Patients met with the surgical team to discuss their treatment recommendations and were given verbal and written information about participating in the study. All patients had an initial consultation with either a medical or clinical oncologist to further discuss treatment options and the study. If the patient expressed a clear preference for or against chemotherapy, such that additional information from the 21-gene assay would not change the decision, then they did not enter the study. For all other patients, after signing an informed consent form, the 21-gene assay was ordered. Once the Recurrence Score results became available, patients had a second consultation with the same oncologist to discuss the results and decide on adjuvant treatment. At both consultations, the patient and the oncologist independently completed a questionnaire (patients: decisional conflict scale (DCS) questionnaire; oncologists: confidence in treatment decisions) (O'Connor, 1993; Holt *et al*, 2013).

### Data analyses

Descriptive statistics were used to summarise clinico-pathological characteristics and Recurrence Score results. McNemar's test was used to assess whether the changes from pre- to post-testing were significant. A paired *t*-test was used to assess the statistical significance of the change in DCS score from pre- to post-testing.

### Ethical approval

The study protocol was approved by the NHS National Research Ethics Service and the R&D Consortia at the participating sites and written informed consent was obtained from participating patients.

## Results

### Patient and tumour characteristics

The final analysis included 137 patients out of 147 recruited patients (3 were excluded/lost to follow up, 6 patients made definitive treatment decisions without considering the Recurrence Score results and 1 patient was inadvertently given a wrong result). The 137 patients included in the current analyses were treated by 10 oncologists. Patient and tumour characteristics are presented in Table 1. The majority of patients (71.5%) were node negative; the most common tumour stage and grade were stage 1 (56.9%) and grade 2 (65.7%). Most patients (96.4%) had ECOG performance status of 0.

The Recurrence Score results ranged between 1 and 76 (median, 17), with 71 patients (51.8%), 58 patients (42.3%), and 8 patients (5.8%) having low (<18), intermediate (18–30), and high (⩾31) Recurrence Score results, respectively (Table 1).

### Recurrence Score results and changes in oncologists' recommendations/patients' choices

Oncologists' treatment recommendations pre- and post-testing were available for 135 patients. Pre-testing, 69 patients (51.1%) were recommended chemohormonal therapy (CHT) and 66 (48.9%) were recommended hormonal therapy (HT) alone. After the Recurrence Score results became available, 55 patients (40.7% 95% confidence interval (CI), 32.3–49.1%) had a change in their treatment recommendation. Of the 69 patients with a pre-testing CHT recommendation, 43 patients (62.3% 95% CI, 50.6–74.0%) had a recommendation change to HT only. Of the 66 patients with a pre-testing HT recommendation, 12 patients (18.2% 95% CI, 8.6–27.7%) had a recommendation change to CHT. These changes led to a net reduction in the oncologists' CHT recommendation rate from 50.4 to 27.7% (*P*<0.0001; McNemar's test).

Overall, the patterns of change in patients' choice of therapy were similar to those observed for the recommendations by the oncologists. Of the 131 patients with treatment choices pre- and post-testing, prior to the availability of the Recurrence Score result, 52 (39.7%) chose CHT and 79 (60.3%) chose HT alone. After the results became available, 41 patients (31.3% 95% CI, 23.3–39.3%) changed their treatment choice. Of the 52 patients with an initial CHT choice, 28 patients (53.8% 95% CI, 39.8–67.9%) changed their choice to HT only. Of the 79 patients with an initial HT choice, 13 (16.5% 95% CI, 8.1–24.8%) changed their choice to CHT. These changes led to a net reduction in CHT use from 39.7 to 28.2% (*P*=0.019; McNemar's test).

### Changes in oncologists' recommendations/patients' choices by Recurrence Score category

**Figure 1:**
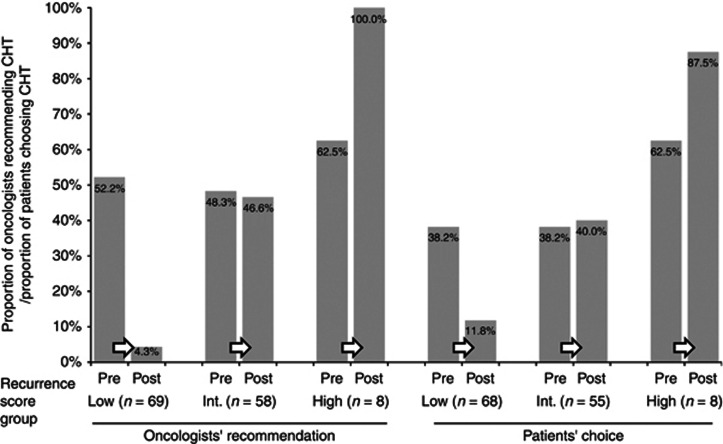
**The impact of knowing the Recurrence Score result on oncologists' recommendations and patients' choice of therapy by Recurrence Score category.** CHT, chemohormonal therapy.

The direction of change in oncologists' recommendations and patients' choices were consistent with the Recurrence Score results (net decrease in chemotherapy recommendation rate in low Recurrence Score patients and net increase in high Recurrence Score patients) (Figure 1). Notably, in the intermediate Recurrence Score category, where the CHT recommendation rate/patient's choice of CHT remained almost unchanged after testing (Figure 1), oncologists and patients did change their recommendations/choices, albeit in similar rates in both directions (i.e., oncologists changed their recommendation from HT to CHT for 9 patients and from CHT to HT in 10 patients; 8 patients changed their choice of treatment from HT to CHT and 7 from CHT to HT).

### Changes in oncologists' recommendations/patients' choices in the subgroup of patients meeting the NICE criteria

**Figure 2:**
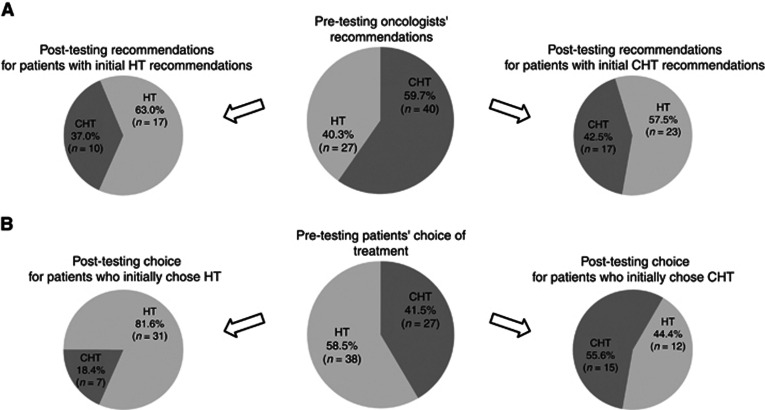
**Impact of knowing the Recurrence Score result on adjuvant treatment in the subgroup of patients meeting the NICE criteria for testing.** (**A**) Oncologists' recommendation for treatment before and after testing (*P*=0.023; McNemar's test). (**B**) Patients' treatment choice before and after testing (*P*=0.25; McNemar's test). CHT, chemohormonal therapy; HT, hormonal therapy.

Following the publication of the NICE DG10, an unplanned retrospective analysis was performed for patients who, using this guidance, were considered at intermediate risk of recurrence using NPI. Sixty-seven patients met these criteria and had oncologists' recommendations pre- and post-testing. Recommendation changes were reported for 33 patients (49.3% 95% CI, 37.0–61.5%); including 10 patients of 27 (37.0% 95% CI, 17.6–56.5%) with an initial HT recommendation and 23 of 40 (57.5% 95% CI, 41.5–73.5%) with an initial CHT recommendation (Figure 2A). The net result was a statistically significant decrease in the CHT recommendation rate from 59.7% to 40.3% (*P*=0.024; McNemar's test). Similar changes were observed in patients' treatment choices, although the magnitude of change was attenuated. Of the 65 patients meeting the NICE criteria and having treatment choices pre- and post testing, 19 patients (29.2% 95% CI, 17.9–40.6%) changed their treatment choice including 7 of 38 (18.4% 95% CI, 5.5–31.3%) with an initial HT choice and 12 of 27 (44.4% 95% CI, 24.4–64.5%) with an initial CHT choice (Figure 2B). The net result was a numerical decrease in the CHT recommendation rate from 41.5% to 33.8% (*P*=0.25; McNemar's test).

### Concordance between oncologists' recommendations and patients' treatment choices

Post-testing, in total, there were 14 cases (10.2%) for which the oncologist's recommendation and the patient's choice of treatment were discordant. In seven cases (5.1%), the oncologist recommended CHT and the patient chose HT, and in another seven cases (5.1%), the discordance was in the reverse direction. The majority of discordant cases (9 cases; 64%) were patients with intermediate Recurrence Score results (Table 2). The patients who chose CHT after they were recommended HT alone were younger (median (range) of 51 (39–57) years), and more likely to have Nmic (three patients, 43%) or N1 (one patient, 14%) disease. The patients who chose HT alone after they were recommended CHT were older (64 (51–69) years) and none had Nmic or N1 disease.

### Recurrence Score testing, oncologists' confidence in treatment decisions and patients' decisional conflict

Knowing the Recurrence Score results improved oncologists' confidence in their treatment recommendations and decreased patients' decisional conflict about their treatment choices. Before testing, oncologists agreed or strongly agreed with the statement ‘I am confident in my treatment recommendations' in 66 of 135 cases (48.9%); whereas, after testing oncologists agreed or strongly agreed with this statement in 109 of 134 cases (81.3%). Patients' decisional conflict decreased after knowing the Recurrence Score result (Table 3). The total DCS score decreased significantly after knowing the test result, as did all the DCS sub-scores (*P*⩽0.0001; *t*-test), except for the support sub-score (*P*=0.067; *t*-test; Table 3; see Supplementary Table S1 for information on patients' responses to individual statements within each subscale).

## Discussion

Our study demonstrated that the 21-gene assay significantly influenced treatment recommendations overall and in the subgroup of patients meeting the NICE criteria with a favourable impact on both physicians' confidence and patients' decisional conflict. These results are consistent with other prospective decision impact studies conducted worldwide (Lo *et al*, 2010; Albanell *et al*, 2012; Davidson *et al*, 2012; de Boer *et al*, 2013; Eiermann *et al*, 2013; Holt *et al*, 2013; Yamauchi *et al*, 2014; Gligorov *et al*, 2015). Our results are also consistent overall with a recent UK study performed by Holt and colleagues comparing pre-testing treatment recommendations (taking into account both the patients' and oncologists' views) and final treatment decisions (post-testing) in 142 women with ER+ node-negative or pN1mic breast cancer where the overall change rate was 27% and chemotherapy use decreased from 40.1% to 30.3%. The Holt *et al* (2013) study demonstrated that testing decreased the total DCS score (and two sub-scores, the informed sub-score and the uncertainty sub-score).

This is the first study to separately evaluate decision impact in patients meeting the recent NICE guidance using NPI>3.4 as a proxy. We showed that in this group, testing had a significant influence on oncologists' treatment recommendations, with a net reduction in the chemotherapy recommendation rate (from 59.7% to 40.3%). The clinical relevance of testing in intermediate-risk (by clinico-pathological parameters) patients has also been demonstrated in a pooled analysis of four European prospective studies where the impact of testing on clinical decisions was shown across tumour sizes and tumour grades (Albanell *et al*, 2013). Interestingly, in this subgroup of patients meeting NICE guidance, the impact on patients' choice of treatment was less profound (compared with the impact on oncologists) and was not statistically significant, suggesting that educating patients about chemotherapy benefit as a function of the Recurrence Score result is warranted.

Our findings from this real-world study suggest that implementing the NICE guidance for using the test could substantially alter treatment patterns and reduce the use of adjuvant chemotherapy for ER+ HER2− node-negative patients across the United Kingdom. A cost-effectiveness analysis performed by Holt *et al* (2013), which was not restricted to intermediate-risk patients, demonstrated an incremental cost-effectiveness ratio (ICER) of £6232 per quality-adjusted life year (QALY) gained in comparison with clinical practice. Furthermore, a probabilistic sensitivity analysis demonstrated 99.6% probability for cost-effectiveness compared with current UK clinical practice assuming a willingness-to-pay threshold of £20 000 per QALY gained (Holt *et al*, 2013). More recent analyses conducted by NICE, which focused on the subpopulation with NPI>3.4 and incorporated the predictive benefits described in the validation study by Paik *et al* (2006), yielded an ICER of £9007 per QALY gained, and a probability of 91.6% of being cost-effective compared with current clinical practice (NICE, 2013). Monitoring the implementation of the NICE guidance (following the UK access theme launched in April 2015) and its impact on clinical practice, clinical outcomes, and the NHS budget is warranted. The NICE guidance is restricted to patients with node-negative intermediate-risk breast cancer. This study suggests that for patients with micrometastases and women over 50 years of age with 1–3 positive axillary lymph nodes, there is similar confidence in the Recurrence Score result influencing chemotherapy decision making by patients and clinicians. Further prospective studies, with cost-effectiveness analyses, are required to confirm this.

This study has several limitations. Although the study cohort was relatively large, the subgroup of patients meeting the NICE criteria was limited and further subgroup analyses could not be conducted (e.g., by age group, size, and grade of cancer). The study was designed to evaluate the impact of the Recurrence Score result on chemotherapy decisions but did not prospectively analyse clinical outcomes in these patients.

In conclusion, in the United Kingdom, knowing the Recurrence Score result reduced chemotherapy use in ER+ HER2− early breast cancer overall, and in the subgroup of patients meeting the NICE criteria, suggesting that implementing NICE guidance could substantially alter treatment patterns in the United Kingdom.

## Figures and Tables

**Table 1:** Baseline patient and tumour characteristics (*N*=137)

**Characteristic**	**Value**
**Age**	
Median (range), years	55 (31–80)
**Tumour stage,** ***n*** **(%)**	
T1	78 (56.9)
T2	55 (40.1)
T3	4 (2.9)
**Tumour grade,** ***n*** **(%)**	
Grade 1	8 (5.8)
Grade 2	90 (65.7)
Grade 3	39 (28.5)
**Nodal status,** ***n*** **(%)**	
N0	98 (71.5)
N1_mic_	11 (8.0)
N1	26 (19.0)
Unknown^a^	2 (1.5)
**Vascular invasion,** ***n*** **(%)**	
Absent	111 (81.0)
Present	26 (19.0)
**Performance status (ECOG),** ***n*** **(%)**	
0	132 (96.4)
1	5 (3.6)

Abbreviation: ECOG=Eastern Cooperative Oncology Group.

a Two patients did not undergo axillary surgery due to previous axillary dissection.

**Table 2:** Post-testing discordance between oncologists' recommendations and patients' choice of therapy by Recurrence Score category (14 discordant cases in total)

**Oncologists' treatment recommendation**	**Patient's treatment choice**	**Recurrence Score category**	**Cases identified,** ***n***
CHT	HT	Intermediate	6
CHT	HT	High	1
HT	CHT	Low	4
HT	CHT	Intermediate	3

Abbreviations: CHT=chemohormonal therapy; HT=hormonal therapy.

**Table 3:** Summary of decisional conflict results; *P*-values were calculated using paired *t*-test

	***n***	**Pre-testing mean**	**Post-testing mean**	**Mean change**	***P*****-value**
Uncertainty subscore	132	40.8	19.1	−21.5	<0.0001
Informed subscore	132	17.7	11.4	−6.4	0.0001
Clarity subscore	132	20.3	12.9	−7.4	<0.0001
Support subscore	136	12.4	9.8	−2.7	0.067
Effective decision subscore	128	19.8	10.3	−9.7	<0.0001
Total DCS score	132	22.1	12.7	−9.5	<0.0001

Abbreviation: DCS=decisional conflict scale.

Lower values indicate less decisional conflict.
