# Supplementary material for: The impact of the 21-gene assay on adjuvant treatment decisions in oestrogen receptor-positive early breast cancer: a prospective study
Source: Br J Cancer. 2016 Mar 8;114(7):731–6. doi: 10.1038/bjc.2016.48 (PMC4984867; doi:10.1038/bjc.2016.48)
Supplement: Supplementary Table S1 [file bjc201648x1.docx]

**Supplementary Material**

**Table S1.** Patients' decisional conflict before and after knowing the Recurrence Score result. Scale is from 1 (strongly agree) to 5 (strongly disagree).

| **Subscale** | **Statement** | **Pre/**  **Post testing** | **Number of patients responding to each statement** | **Proportion of patients responding** | | | | |
| --- | --- | --- | --- | --- | --- | --- | --- | --- |
|  |  |  |  | **1** | **2** | **3** | **4** | **5** |
| **Uncertainty subscale** | This decision is easy for me to make | pre | 136 | 29 | 23 | 11 | 22 | 15 |
|  |  | post | 132 | 52 | 23 | 10 | 9 | 6 |
|  | I’m sure what to do in this decision | pre | 137 | 28 | 27 | 15 | 19 | 12 |
|  |  | post | 132 | 51 | 33 | 8 | 5 | 3 |
|  | It’s clear what choice is best for me | pre | 137 | 28 | 26 | 19 | 18 | 10 |
|  |  | post | 132 | 61 | 24 | 11 | 2 | 2 |
| **Informed subscale** | I’m aware of the options I have in this decision | pre | 137 | 56 | 42 | 0 | 2 | 2 |
|  |  | post | 132 | 69 | 30 | 0 | 0 | 1 |
|  | I feel I know the advantages of each option | pre | 137 | 37 | 53 | 7 | 1 | 2 |
|  |  | post | 132 | 56 | 41 | 2 | 1 | 1 |
|  | I feel I know the disadvantages of each option | pre | 137 | 34 | 53 | 10 | 2 | 2 |
|  |  | post | 131 | 50 | 46 | 3 | 0 | 1 |
| **Clarity subscale** | I am clear about how important the advantages are to me in this decision | pre | 137 | 43 | 47 | 8 | 0 | 2 |
|  |  | post | 132 | 64 | 33 | 2 | 0 | 1 |
|  | I am clear about how important the disadvantages are to me in this decision | pre | 136 | 36 | 49 | 13 | 0 | 2 |
|  |  | post | 129 | 47 | 47 | 5 | 0 | 2 |
|  | For the main options I am considering, I am clear about which is more important to me (the advantages or disadvantages) | pre | 136 | 33 | 51 | 12 | 2 | 2 |
|  |  | post | 129 | 57 | 37 | 5 | 1 | 1 |
| **Support subscale** | I am making this choice without any pressure from others | pre | 136 | 60 | 35 | 4 | 1 | 1 |
|  |  | post | 129 | 69 | 24 | 5 | 2 | 1 |
|  | I have the right amount of support from others in making this choice | pre | 136 | 64 | 33 | 2 | 0 | 1 |
|  |  | post | 129 | 68 | 26 | 4 | 1 | 1 |
|  | I have enough advice about the options | pre | 136 | 46 | 48 | 5 | 0 | 1 |
|  |  | post | 129 | 67 | 30 | 2 | 0 | 1 |
| **Effective decision subscale** | I feel I have made an informed choice | pre | 134 | 46 | 39 | 13 | 2 | 2 |
|  |  | post | 128 | 68 | 29 | 2 | 0 | 1 |
|  | My decision shows what is important to me | pre | 134 | 47 | 41 | 11 | 0 | 1 |
|  |  | post | 129 | 64 | 32 | 4 | 0 | 1 |
|  | I expect to stick with my decision | pre | 136 | 38 | 39 | 21 | 2 | 1 |
|  |  | post | 129 | 70 | 23 | 7 | 0 | 1 |
|  | I am satisfied with my decision | pre | 135 | 40 | 36 | 22 | 1 | 2 |
|  |  | post | 129 | 64 | 30 | 5 | 1 | 1 |
